# Long-latency auditory evoked responses across species show increased amplitude during early life

**DOI:** 10.1093/cercor/bhaf274

**Published:** 2026-01-07

**Authors:** Krista Lehtomäki, Jari Keinänen, Riaz Uddin Mondal, Lauri Parkkonen, Markku Penttonen, Tiina Parviainen

**Affiliations:** Center for Interdisciplinary Brain Research & Department of Psychology, Alvar Aallon katu 9, University of Jyväskylä, 40014 Jyväskylä, Finland; Center for Interdisciplinary Brain Research & Department of Psychology, Alvar Aallon katu 9, University of Jyväskylä, 40014 Jyväskylä, Finland; Department of Information & Communication Engineering, Faculty of Engineering, East Block, 4th Science Building, University of Rajshahi, Rajshahi 6205, Bangladesh; Faculty of Information Technology, Mattilanniemi 2, University of Jyväskylä, 40014 Jyväskylä, Finland; Department of Neuroscience and Biomedical Engineering, School of Science, Otakaari 3, Aalto University, 00076 Espoo, Finland; Center for Interdisciplinary Brain Research & Department of Psychology, Alvar Aallon katu 9, University of Jyväskylä, 40014 Jyväskylä, Finland; Center for Interdisciplinary Brain Research & Department of Psychology, Alvar Aallon katu 9, University of Jyväskylä, 40014 Jyväskylä, Finland

**Keywords:** auditory evoked responses, cross-species, ECoG, electrocorticography, electrophysiology, juvenile, magnetoencephalography, MEG

## Abstract

Auditory evoked responses undergo notable changes during childhood, likely reflecting modifications in synaptic signaling in the auditory cortex. Particularly robust response, observed around 200 to 300 ms post stimulus (N/M250), has been consistently reported in children but is absent in adults. This long-latency response, evoked even in passive listening conditions, may indicate heightened sensory pathway responsiveness, facilitating experience-driven cortical plasticity. However, it remains unclear whether this delayed activation pattern is an intrinsic, species-general feature of brain development. We recorded cortical auditory evoked responses to monaural sine-wave tones/click sounds in 3 age groups (preadolescents, adolescents, and young adults) of human subjects and rats. Following short-lived early responses, both species exhibited a long-latency (150 to 450 ms) response in the auditory cortex. In both species, the relative amplitude of the long-latency response, compared to early responses, was increased in younger individuals. In human children, single-trial analysis demonstrated more consistent trial-by-trial timing of the response in this later time window than in the adult-typical 100-ms response in the earlier time window. Given its emergence in purely passive conditions, and across species, the robust current activity in late time window could represent a distinct synaptic event and may serve as a marker of the maturational stage, particularly in GABAergic cortical circuits.

## Introduction

Cortical development proceeds as an interplay between genetically driven maturational processes and environmental influence. The nervous system is modified remarkably during sensitive periods in early life (Johnson 2001; Johnson 2005). This capacity of cortical plasticity underlies the adaptation of the brain’s wiring in response to the unique growth environment, thus providing the basis for a mature brain network and behavior. Despite its essential role in structuring the cerebral cortex, surprisingly little is known about the developmental trajectory of brain–environment interactions or, more specifically, the reactivity of cortical circuitry to external stimulation, especially in humans.

Research on auditory perception in humans and nonhuman species that utilize hearing in social communication provides insights into our understanding of the development of behaviorally meaningful neural processes. In humans, auditory cortex electrophysiology shows notable changes across development in noninvasively recorded electrophysiological signals from outside the skull (Albrecht et al. 2000; Takeshita et al. 2002; Wunderlich and Cone-Wesson 2006). In animal models, it has been possible to scrutinize the neurophysiological changes at the level of cortical circuitry across age using invasive recordings (Takesian and Hensch 2013), but there is no research data on the extracortical response pattern in the juvenile stage. We aimed to determine whether the developmentally specific features of auditory responses represent general characteristics of the developing circuitry by implementing parallel stimulation paradigms across maturational stages in humans and rats.

The auditory system converts the air pressure changes received by the ear into patterns of neural activity that ultimately support all auditory-based behavior in humans and other species. The sequence of deflections in the auditory evoked response (AER), measured by time-sensitive neuroimaging methods such as electroencephalography (EEG) and magnetoencephalography (MEG), reflects this multiphase processing in the brain (Picton et al. 1974; Hari 1990). The neural computation underlying these externally measured responses can be quantified and extrapolated by the timing, amplitude, polarity, ie the direction of the underlying neural current, and spatial distribution of individual deflections. Automatic (obligatory) responses to auditory input arise ~ 0 to 200 ms after the onset of a sound and, similarly to early somatosensory responses (Stephani et al. 2021), can be seen to reflect mostly physical characteristics of the stimulus. Developmental changes in these responses have been suggested to reflect development of interlayer and intracortical connections and synaptic maturation (Eggermont and Ponton 2003) as well as the excitability level of the cortex (Bruyns-Haylett et al. 2017). Indeed, simple auditory stimulation provides a means to examine the reactivity properties of neural circuitry along the auditory pathway. Specifically, cortical responses to simple stimuli, especially under passive stimulation conditions, may reveal the structural characteristics of the processing pathway. For example, response latencies and spatial distribution are directly shaped by the micro- and macro-level connectivity patterns of the cortex.

The early auditory brainstem responses at 1 to 10 ms post stimulus (Johnson et al. 2005) are followed by middle-latency responses at ~10 to 60 ms (Özdamar and Kraus 1983; McGee and Kraus 1996), which are generated by various thalamocortical brain structures (Kraus and McGee 1992). Characteristic of the later auditory responses, roughly after 100 to 200 ms, is that while they signify the cortical processing sequence engaged in processing any auditory input, they also associate with perceptual and cognitive operations (Picton 2011). Indeed, already at 100 ms, source modeling of the underlying generators of the evoked activity suggests the origin of the responses to be in synaptic signaling within the nonprimary auditory area, centering in Heschl’s gyrus or the adjacent planum temporale (Parviainen et al. 2005). The most prominent adult-age deflection at ~100 ms (N/M100) emerges surprisingly late developmentally, by the age of 9 years (Cunningham et al. 2000; Kraus et al. 1993), although at a somewhat younger age with a longer interstimulus interval (Ceponiene et al. 2002; Takeshita et al. 2002). Despite late emergence, it shows similar spatial coordinates to those in adults (Parviainen et al. 2011). The developmental pattern of early responses, particularly the P50 response, has been well characterized, demonstrating delayed latencies in children (~100 ms) and a systematic age-related decrease in latency (Ponton et al. 2000; Sharma et al. 2002; Eggermont and Ponton 2003). This response often overlaps with the emerging N100 response in surface recordings, which can complicate group-level interpretations, particularly in children under 10 years of age.

Of particular interest for maturational changes and plasticity is the robust response evidenced throughout childhood at ~200 to 300 ms (referred to in the EEG/MEG literature as the N/M250 response), which is not reported in adult studies. Previous studies in children have systematically reported this long-lasting and late obligatory response at ~250 ms post-stimulus in response to the passive presentation of simple sounds (Cunningham et al. 2000; Sussman et al. 2008). It appears to reflect generators distinct from those underlying the developmentally later-emerging N/M100 (Parviainen et al. 2019; 2011) and the adult N2, which is observed only in active paradigms and associated with cognitive processes such as attentional control (Huster et al. 2010; van Bijnen et al. 2022). The N/M250 response seem to dominate during childhood, and represent the prominent obligatory responses component in children, but its amplitude has been suggested to decrease to adult values by the age of 17 (Ponton et al. 2000). Given electric activity in this time window in adults is typically linked with cognitive (ie “higher-level”) information processing, it is somewhat surprising that children show current activity in this time window in purely passive paradigms. Interestingly, despite the obligatory nature of this response, its amplitude has been associated with the cognitive and behavioral competence, such as efficiency of information processing (Ceponiene et al. 2002; Takeshita et al. 2002), and response inhibition (van Bijnen et al. 2022) both in context of typical (Parviainen et al. 2011) and atypical development (van Bijnen et al. 2019). This long-latency current activity could thus reflect a general, but behaviorally meaningful feature of developing (auditory) cortex.

Some evidence suggests that a delayed and prolonged activation pattern can be observed in other sensory modalities of a developing brain as well. Both somatosensory cortical responses to tactile stimulation (Pihko et al. 2009) and transcranial magnetic stimulation (TMS)-evoked EEG responses in the motor cortex (Määttä et al. 2017) exhibited a sluggish late deflection in the developing brain, with similar timing to the late auditory response at 200 to 300 ms, in comparison to adults who evidence prominent early responses with no further distinctive activation. In general, since the later time window is more likely to reflect the stage of cortical processing related to the integration of input across sensory modalities, or top-down influence on sensory processing, it is not surprising that responses to stimuli in different modalities would exhibit similar age-related characteristics. Hypothetically, it would make sense that developing sensory pathways demonstrate stronger and longer-lasting responsivity to the external input, potentially benefitting the experience-driven modification of the cortical circuitry. So far, there have been no attempts to resolve whether the delayed activation pattern represents an intrinsic, species-general feature in the developing brain.

In rodent models, interest is usually in detailed cellular and laminar level functions, and it is not a common practice to measure cortical potentials, which is the usual measurement modality for the human brain. Electrophysiological responses measured from the cortex are considered a rather inexact indicator as they sum the electric activity over various events in the underlying cortical circuitry. To reach a more detailed understanding of the developmental processes in the human cortex, it is necessary to determine the correspondence in cortical phenomena between human and other mammalian (model) species that show circuitry properties similar to those of humans. Although comparisons across species have been made at the general level, the measurement protocols and techniques are typically not harmonized to enable unified interpretations. Here, we used auditory stimuli to record the electrophysiological response properties of the auditory cortex in humans and rats at different ages across their developmental trajectories.

AERs to simple sound stimuli have been measured earlier from the mature rat’s primary auditory cortex (A1) (Barth and Di 1990; Takahashi et al. 2005). When comparing the human auditory system to that of other mammals, the range of frequency sensitivity or threshold levels differs, but cochlear mechanics and the generation of electrical activity function according to similar principles (Eggermont and Odenthal 1974; Kraus and McGee 1992). The smaller size of a rat’s brain results in faster conduction time and shorter latencies in response patterns compared to humans, but the principles and time-constants involved in synaptic signaling are preserved across mammalian species (Barron et al. 2021). With regression analysis focusing on AERs Sambeth et al. (2003) concluded a 1.8 multiplication factor between the corresponding components in rats vs. humans, particularly valid for shorter latencies. In line also with other reports (Mahmoudzadeh et al. 2017; Shinba 1997), the well-known auditory P50 response in humans is considered to correspond to the response occurring at 10 to 30 ms in rats. In the same way, the counterpart of the N100 response is contemplated to occur at ~41 to 80 ms in rats, P200 at 80 to 130 ms (sometimes up to 200 ms), N200 at 130 to 200 ms (sometimes up to 250 ms), and P300 at 250 to 500 ms. The estimates, however, vary somewhat across studies. Importantly, no studies have reported auditory cortical responses in rats during the juvenile stage.

The unquestionable benefit of cortical evoked responses is that, when probed by simple stimuli, they provide accurate information on the general timing of synaptic activity, with high temporal resolution, and are also easily accessible in humans. Indeed, rather than focusing on individual responses or components, it is more meaningful to consider these deflections collectively as an evolving wave of electrical activity in the cortical pathway. The progression of bulk electrical currents reflected in the waveform can be interpreted as representing the computational steps along the auditory pathway(s).

It is conventional to focus on the averaged evoked responses with the underlying assumption that there is a standard, unchanged sequence of neural responses (hence, local increases in current activity) to individual stimulus presentations, and that the trial-by-trial variation can be ignored as noise. However, it may well be that—compared to the adult brain—the developing brain shows larger variance in timing of individual responses, which feature would not be captured by comparing average responses across ages. The improved accuracy in timing of synaptic signaling has been suggested as one of the core mechanisms that change along with development (Oswald and Reyes 2011), and as synaptic currents underlie the evoked response characteristics, it is essential to explore the single-trial characteristics of evoked responses to more correctly interpret system-level signatures of cortical development. Indeed, a prolonged increase in amplitude in the averaged waveform (characterizing the M250 response; Parviainen et al. 2011; Ruhnau et al. 2011; Parviainen et al. 2019), could in principle reflect jittered single-trial responses (with high enough single-trial amplitudes) that *on average* have the latency of the 250 ms response. An alternative explanation is that every single trial across the experiment shows this prolonged response. These 2 alternatives assume very different underlying cause for age-related variation. Clarifying the trial-by-trial variance in timing of the prolonged response will enable us to determine whether the neurophysiological characteristics underlying the maturational changes indicate differences in the temporal precision of synaptic signaling across trials or specific, temporally distinct synaptic event(s).

This study aimed to resolve whether the late activation pattern in the auditory system is a general feature of the developing brain and can be evidenced across mammalian species. Based on the previous studies on the auditory and other sensory systems, it is reasonable to assume that the delayed juvenile-specific pattern of activity could be an evolutionarily preserved phenomenon, featuring increased reactivity of the cortex by sensory stimulation. We explored whether juvenile rats and young human subjects, respectively, show similar age-related differences in the pattern of auditory activation, specifically focusing on the relative emphasis on early current activity at ~100 ms vs. late responses at ~200 to 300 ms post stimulus. The evoked activity to simple auditory probes is expected to show similar emphasis on long-latency prolonged response in juvenile animals and children, and the amplitude of this response is expected to decrease with age. Furthermore, we explore the trial-by-trial variance in response amplitude and latency in both species to clarify whether the prolonged response pattern is detectable at single trials (indicating distinct synaptic event) and to reveal if juvenile brain features increased variance/decreased temporal acuity. We expect to see the age-dependent prolonged pattern at the level of single trials, ensuring that the differences relate to the prolonged reactivity of the cortical circuitry rather than imprecise timing.

## Materials and methods

### Subjects

#### Humans

A total of 11 adults (aged 19 to 26 yr, 6 females) and 23 children (aged 9 to 13.5 yr, 13 females) were recruited via university mailing lists and from schools in the Oxford area in the United Kingdom. Participants were required to speak English as their mother tongue, be right-handed, have normal hearing, and have no history of neurological or psychiatric diseases. Children were divided into 2 age groups: 9 to 10.5 yr (preadolescents, *n* = 11, 6 females) and 12 to 13.5 yr (adolescents, *n* = 11, 7 females). 19 to 26-year-old adults formed the “young adults” group (*n* = 11, 6 females). One outlier in the adolescent group was excluded from the data since all amplitude values for all measures were 1.5 interquartile ranges above the third quartile. All adult participants gave informed consent before taking part to the experiment. In the case of children, their parents gave the informed consent. Participants were reimbursed for the expenses incurred by their participation. The MEG measurements took place at the Oxford Centre for Human Brain Activity at the Warneford Hospital in Oxford. The study was approved by the Central University Research Ethics Committee, University of Oxford (MSD/IDREC/C1/2010/52). The presented study is part of a larger research project on spoken language perception in the developing brain. The follow-up design with rodents was planned for the aim of comparison across species. Therefore, we aimed to take into account the design of the human experimental paradigm when designing the detailed parameters for the rats. However, due to different species, it was not expedient to apply exactly the same parameters.

#### Rats

Cross-sectional rodent data from Wistar laboratory rats aged 28 to 95 postnatal days were collected at the University of Jyväskylä, Finland, to enable comparative analyses of juvenile-specific responses and age-related differences in auditory processing across species. Mature rats were ordered from Envigo, Denmark, and pregnant female rats were ordered from Kuopio, Finland. Juvenile rats were born and raised at the Laboratory Centre of the University of Jyvaskylä. The animals were housed in small groups in metal cages, fed ad libitum, and kept under a 12-hour light–dark cycle with lights on at 7 AM and off at 7 PM. For the analysis, the rats were divided into 3 subgroups corresponding to the age groups of humans. Juvenile rats were classified into three developmental stages: preadolescents (28–42 postnatal days; n = 10, 4 females; Freitas et al. 2002), adolescents (43–64 postnatal days; n = 10, 3 females; Stumpp et al. 2006), and young adults (65–95 postnatal days; n = 10, 3 females; Crews et al. 2000) See Table 1.

**Table 1:** Demographic data of human subjects and rats. Age in days (rats) and in years (humans), number of individuals, mean weight (rats, in grams), and the species’ general maturational stage for the 3 different groups.

	**Rats**	**Humans**
Preadolescents	28 to 42 postnatal days *n* = 10 Weight, *M* = 90.2 g	9 to 10.5 yr *n* = 11
Adolescents	43 to 64 postnatal days *n* = 10 Weight, *M* = 185.2 g	12 to 13.5 yr *n* = 11
Young adults	65 to 95 postnatal days *n* = 10 Weight, *M* = 250.3 g	19 to 26 yr *n* = 11

The experimental procedures for rodents used in this study were approved by the Regional State Administrative Agency for Southern Finland (permit number ESAVI/7762/04.10.07/2016). Experiments were carried out in accordance with the European Communities Council Directive (86/609/EEC) regarding the care and use of animals for experimental procedures. All the researchers who conducted the animal experiments received special training on the care and treatment of laboratory animals, which is mandatory for using nonhuman vertebrates for research purposes. The ethical problems were discussed in terms of the 3 Rs (reduction, refinement, and replacement). In terms of reduction, the number of animals was adjusted so that it was possible to obtain reliable results with a minimal number of animals. In the spirit of the principle of refinement, a within-subjects design was used whenever possible. In addition, the experiments were designed so that the results answered more than 1 question at a time. The principle of replacement manifested in our studies as we conducted experiments in animals only when we could not answer the questions of developmental plasticity in human experiments.

### Stimuli

The aim of the cross-species comparison was to determine whether both species show a developmental difference in the auditory activation by simple stimulation—therefore, the main comparison was done between early and late time windows “within” species. Simple passively presented auditory stimuli were used in the recordings of both species. It was important to optimize the stimulation protocol for the given species, but at the same time to ensure the alignment of the general response properties that are most likely influenced by temporal characteristics of stimulus presentation.

In human measurements, the stimuli were 1-kHz pure tones with a duration of 50 ms and a 15-ms rise and fall times, created in Adobe Audition 1.5 (Adobe Systems Incorporated, CA, USA). A total of 205 tones were presented monaurally to the left and right ears, alternately between the ears via inserted earphones, using Presentation software (Neurobehavioral System Inc., San Francisco, CA). The interstimulus interval (ISI) was randomized between 0.8 and 1.2 s, creating an ISI of 1.6 to 2.4 s for each ear. The individual auditory threshold was obtained for each subject to ensure that all subjects had normal hearing, and during the MEG recordings, the tones were presented 65 dB above hearing level. Only right-ear sounds were used in the analysis.

The stimuli in animal measurements were guided by those used in human recordings while acknowledging species-specific properties of the auditory system. Thus, the stimuli in the animal experiment were pure tones and short clicks (duration of 1 ms), all presented to the right ear at 75 dB (ensuring audibility under anesthesia). Tones were 6 to 15 kHz sounds (duration of 100 ms with the 8-ms rise and fall times), aligning with species relevant auditory characteristics, output from the computer sound card, and amplified with the SA1 speaker amplifier (TuckerDavis Technologies, Alachua, FL, USA). The sound intensity was measured with Bruel & Kjaer precision sound level meter type 2235 using linear weighting, peak intensity 2 kHz. Altogether 40 pure tones (frequencies of 3, 6, 9, and 15 kHz) and 70 click sounds were presented, in separate series, with a random ISI of 1.6 to 2.4 s for both pure tones and click sounds matching with the human recordings. More specifically, the interstimulus interval of the rat measurements was set to be twice as much as that of the human measurements, 1.6 to 2.4 s in rats and 0.8 to 1.2 s in humans, so that the ISI was the same from the perspective of right-ear stimulation. While in humans the stimulation was binaural, with left and right ear stimuli alternating, in rats we used monaural right-ear stimulation due to the use of stereotaxical stabilization with an ear bar installed in the left ear. The presentation of the sounds was programmed using E-prime, version 1.0 (Psychology Software Tools, Sharpsburg, USA). The program also generated TTL pulses to locate the start and type of each stimulus. The pulses were used to generate pulses of different amplitudes with a D/A converter to represent different stimulus types.

In humans, we used sine-wave tones as stimuli; and in rats, both sine-wave tones and click sounds were used. Because the click sounds evoked better-quality responses in rats, they were used for statistical analysis instead of sine-wave tones. However, sine-wave tones (which were used in humans) gave essentially identical general-level findings in rats. For comparison, Supplementary Appendix A shows the average response to sine-wave tones in rats.

### MEG data acquisition and preprocessing in humans

Magnetic signals were recorded with a 306-sensor whole-head neuromagnetometer (VectorViewTM system; Elekta Neuromag Oy, Helsinki, Finland) in a magnetically shielded room. Measurement sensors are arranged in triple-sensor elements at 122 locations so that every element consists of 2 orthogonally oriented planar gradiometers and 1 magnetometer. A magnetometer is a simple loop that is most sensitive to source currents that surround the loop, while a planar gradiometer with 2 oppositely wound in-plane loops detects the maximum signal directly above an active brain area. The position of each subject’s head relative to the recording sensors was determined using 4 head position indicator (HPI) coils attached to the scalp. Before the measurement, the locations of the coils were registered with a Polhemus Fastrak® digitizer (Colchester, VT, USA). Three anatomical landmarks (nasion, left, and right preauricular reference points) were also digitized to define a head-based MEG coordinate system where the *x*-axis passes through the preauricular points from left to right, the *y*-axis passes through the nasion perpendicular to the *x*-axis, and the *z*-axis points upward. To determine the location of the HPI coils in the MEG-helmet, the coils were briefly activated at the beginning of the recordings. Using the HPI coils, it was also possible to monitor changes in head position throughout the entire measurement. This was applied in 6 children who had difficulties keeping their heads still during the measurements. Horizontal and vertical eye movements (electro-oculogram) were recorded to detect eye movements and blinks that could interfere with the data.

The MEG signals were bandpass filtered at 0.03 to 333 Hz and sampled at 600 Hz. The signal space separation (SSS) method (Taulu and Simola 2006) was applied to suppress interfering signals outside of the MEG sensor array. If continuous head position indicator (cHPI) was used, SSS was applied with movement compensation (in 6 participants). If the SSS-corrected raw MEG data indicated remaining artifacts, as revealed by visual inspection of the data and/or automatic epoching procedure, the temporal extension of SSS (tSSS) was applied (in 5 participants). Head position was estimated with a buffer length of 30 s and a correlation limit of 0.980. The MEG data were averaged offline from 200 ms before to 800 ms after each stimulus onset. Only gradiometers were used, as magnetometers are more sensitive to external noise. The epochs contaminated by eye movements, identified via the EOG signal (exceeding ± 150 μV), were excluded from the average. Additionally, epochs with excessive noise (exceeding 3000 fT/cm) were removed to minimize artifacts. On average, 105 (minimum 76) artifact-free trials per category were gathered from each subject. Prior to further analysis, the averaged MEG responses were baseline-corrected using the 200-ms interval preceding stimulus onset and low-pass filtered at 40 Hz with a Hamming-windowed FIR filter and a 10 Hz transition bandwith.

### Source localization of auditory activation in humans

**Fig. 1:**
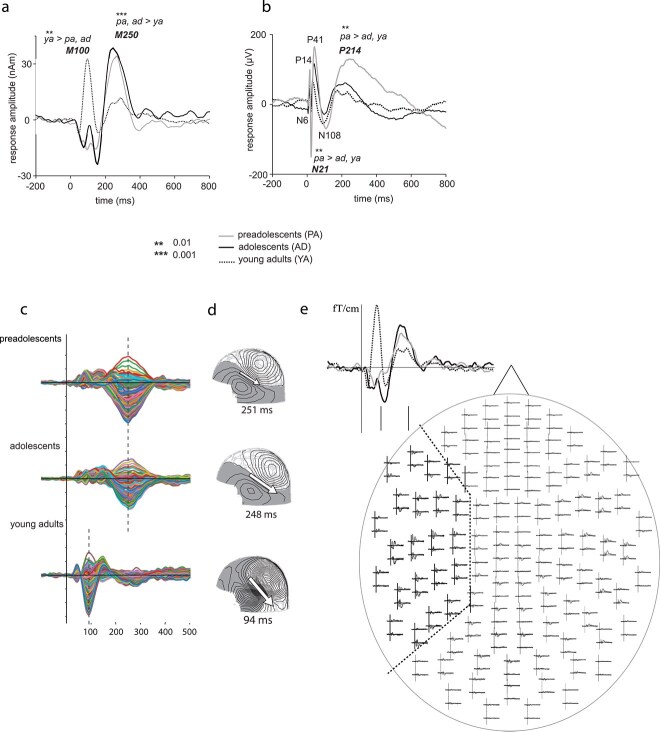
Time course and location of auditory activation in humans and rats. (a) Time course of activation evoked by the auditory probes in the current source located in the left supratemporal auditory cortex in humans. Significant differences between age groups are indicated by asterisks. (b) Time course of activation evoked by the auditory probes as recorded by the silver wire electrode from the surface of the auditory cortex in rats. (c) MNE source waveforms in the left hemisphere ROI (Heschl’s gyrus, Heschl’s sulcus, and planum temporale) shown for each individual voxel, with separate rows representing the 3 different age groups (see Supplementary Appendix C for methods). (d) The location and orientation of the equivalent current dipole (ECD) in humans, fitted at the time point of maximum amplitude for each age group (250 ms for children, 100 ms for adults). (e) Whole-head sensor array with evoked responses in the 3 age groups of humans and 1 sensor enlarged to provide model-free illustration of the timeline of activation. The distribution of sensors used for localizing the ECD is delimited with a dashed line.

To localize the source currents underlying the recorded evoked responses in humans, we used equivalent current dipoles (ECDs) modeled separately for each individual (Hämäläinen et al. 1993). An ECD represents the center of activation and the mean strength and orientation of the electric current in a given brain area. As our focus was on late-emerging activation in children, the time point of maximally dipolar, distinctive field pattern within the general time window around the distinctive 250 ms-response (Fig. 1a) was selected for localizing the current source. In the case that no robust response was present in this time window, the earlier 100 ms activation peak was used for localizing activation. The 100-ms activation in adults and older children shows a highly comparable current location and orientation to the later 250-ms activation (Fig. 1d) and their topographic distribution at channel-level overlaps considerably (Fig. 1e), justifying that the same macro-level model can be used to estimate the amplitude time-course of the underlying current source (see also Parviainen et al. 2011; Parviainen et al. 2019).

To determine the ECDs, a channel selection covering optimally the spatial extent of activation at the sensor level in the left hemisphere was used. As the auditory evoked activation across subjects was systematically evoked within the same general region, a standard set of 22 sensors was used for all individuals (see Fig. 1e). Figure 1d illustrates the current location and orientation in the 3 subject groups. The source model indicated activation generally in the left temporal area, with the current orientation indicating localization to the supratemporal auditory cortex.

The strength and timing of the AERs were collected separately for each subject and for the M250 and M100 responses. In children (both preadolescent and adolescent groups), the most robust activation, detectable in each individual, occurred at ~250 ms after the stimulus onset and in adults, the strongest response was the M100 in the early time window (Fig. 1a and c). This pattern of activity was also evident at the sensor level (ie in the “raw” data) and therefore could not be attributed to the use of a different time window for fitting the ECD. Importantly, we only considered current activity with similar current orientation (current directed downwards/away from the surface of supratemporal plane, typical for M100/M250 response). The ECD source represents the current with a certain location and direction within the cortical laminae. The polarity of the MEG evoked response waveform should be interpreted with reference to the direction of the ECD source. As the amplitude waveform reflects current activity with specific direction (and location), it was not reasonable to extract values from the negative part of the waveform in humans. It is worth noting that the early time window in young children often features a P/M50 response, with current direction opposite to M100 response, which was not quantified in this study.

### E‌EG data acquisition and analysis in rodents

The EEG recordings were made in light anesthesia, known not to influence sensory processing. Corresponding to the human measures of this study, auditory responses from the left hemisphere to right ear sounds were gathered. To begin the operation, the animals were anesthetized with isoflurane (3.5%, 0.9 L/min) in a chamber. Under anesthesia, they were stereotaxically stabilized with nonpuncture ear bars, and isoflurane was applied through an inhalation mask while the concentration was reduced to 2%. Prior to the surgery, urethane was dosed intraperitoneally (1.2 g/kg, concentration 0.24 g/mL, Sigma-Aldrich, St. Louis, MO, USA), and local anesthesia (bupivacaine hydrochloridum, Bupivacaine Accord, 5 mg/mL, Orion, Finland) was applied on the surgery region to ensure the painless operation. The rat was laid on a heated platform (World Precision Instruments, Sarasota, FL, USA), and a temperature controller (ATC2000) was used with a rodent rectal temperature probe to assist in maintaining the body temperature of the animal at 38.2 °C. Isoflurane was cut out 20 min before the acute recordings. During the measurements, the depth of anesthesia was controlled by regular testing of the withdrawal pedal reflex. Supplementing doses of urethane (~0.1–0.2 mL) were applied during the operation if needed to maintain a proper level of anesthesia. Also, 2 mL of saline was added every 2 h for adult rats and ~0.5 mL for juvenile rats to maintain a stable physiological condition.

For the auditory epidural recordings, a craniotomy was conducted. The skin and muscles over the horizontally located skull and the left auditory cortex were removed. The skull was cleaned with cotton buds and hydrogen peroxide and washed with saline. If needed, the bleeding of the muscles was stopped using silver nitrate. The location of the primary auditory cortex (A1) was calculated by using the anatomical point bregma as a landmark. In adults, an opening was drilled to the squamosal bone of the skull: from the bregma anterior–posterior, (−4.5) to (−6.5) mm; and dorsoventral, 3 to 5 mm. In juveniles, the size of the opening was adjusted according to the age of the rat concerning the distance between the bregma and lambda. Two holes were drilled over the frontal cortex, and screws (Ø 1.0 mm) were driven to the bone. Screws were attached to the end of a 10*80 mm Plexiglas plate with acrylic, and the other end of the plate was fixed to the stereotaxic instrument.

For the reference electrode (a 28-gauge stainless steel needle, Ø 0.36 mm, BD Lo-Dose syringe, USA), a 1-mm diameter hole was drilled over the right cerebellum 1.5 mm posterior to the lambda and 2 mm lateral to the midline. The needle was inserted into the hole 1 mm below the surface of the cerebellum and fixed to the skull with cyanoacrylate glue. The ground electrode was a needle similar to the reference electrode and inserted subcutaneously in the neck. A silver wire recording electrode (Ø 0.5 mm, A-M Systems, WA, USA) was positioned on the surface of the dura and attached to a miniature connector together with ground and reference electrodes. The right ear bar was removed, and a speaker (MF1 magnetic speaker, Tucker-Davis Technologies, Alachua, FL, USA) was positioned at the level of the ear and toward it at a distance of 20 cm. After the bar was removed, the head remained stable with the previously fixed acrylic plate.

The recordings were performed in a Faraday cage. A short stimulus test series of click sounds with an ISI of 2 s was played before the acute recordings to ensure that the techniques work properly and that the area of measurement was valid. Large veins were avoided when the electrode was placed. The electrode was positioned over the different parts of the opening, and the location of the largest early responses in the time interval 0 to 50 ms was selected as the position of recording. In most cases, the most optimal measurement point was right in the middle of the opening. At each site, 3 to 5 responses were recorded to evaluate the amplitude of the response. During the recordings, spontaneous and evoked neural activity was monitored online. The signals were initially amplified 10x with the MPA8I head stage and then high-pass filtered at 0.1 Hz, amplified 50x, and low-pass filtered at 5 kHz with an FA16I filter amplifier (all from Multichannelsystems, Reutlingen, Germany). The signals were digitized using an ME64 system (Multichannelsystems) at 20 kHz, low-pass filtered to 600 Hz, downsampled to 2 kHz, and written to a hard disk using the MC_Rack program (V 4.6.2, Multichannelsystems). After the recordings, the anesthetized animals were administered an overdose of general anesthetics by i.p. injection (urethane, Sigma-Aldrich, St. Louis, MO, USA).

The EEG data were analyzed with the BrainVision Analyzer 2.1 program (BrainProducts, Gilching, Germany). Epochs 200 ms before and 800 ms after the stimulus were segmented, visually monitored for artifacts and averaged for each stimulus type. However, no sweeps were rejected from any animals, and no animals were excluded from the final analysis. The averages were written into text files, imported to Excel, and averaged over animals in each age group. A total of 6 auditory responses from rats were analyzed (N6, P14, N21, P41, N108, and P214). The responses were named based on their polarity (positive or negative) relative to the reference electrode and their peak latency (ms). Note, that the polarity is not unequivocally comparable across species due to differences in geometry of the auditory cortex localization in reference to the recording surface. Also, in the rat data polarity is determined by reference electrode, while in human data polarity is determined by the orientation of the ECD fitted to specific time window.

### Single-trial analysis in humans and rodents

**Fig. 2:**
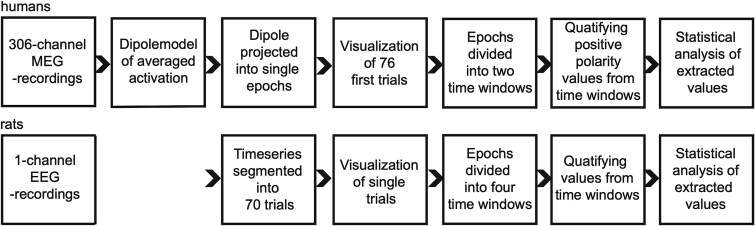
The single-trial analysis pipeline. Analysis steps for extracting the single-trial latency and amplitude values for the MEG recordings in humans and EEG recordings in rats.

Besides the analysis of averaged responses in each individual, the single trials, ie the epochs of electric activation evoked by each sound stimulus, were also analyzed in each subject. Figure 2 shows a pipeline of single-trial analysis in humans and rats. In humans, for extracting single trials, the continuous time-series of the original data was first reproduced at the source level by applying the ECDs obtained from the averaged data by using MNE-Python functions. The single-trial time series were epoched from the continuous data for further quantification of the trial-to-trial consistency of the evoked response amplitude in different time windows. For the electrophysiological recordings of the rat auditory cortex, continuous recording was used directly to extract single-trial epochs.

For analyzing the trial-to-trial variance of the local maxima in the amplitude waveform, the time course of auditory epochs was divided into early (0 to 150 ms) and late (150 to 450 ms) time windows in both humans and rats. In rats, the early time window covered the fast brainstem responses (0 to 10 ms) and the peaks originating in subcortical and early sensory cortical circuits (10 to 30 ms). In MEG recordings in humans the same stimulation settings cannot be used to produce reliable evoked responses both from the brainstem and subcortical or primary cortical structures at the early time window (<50 ms) and from nonprimary auditory areas at the later (>50 ms) time window, and the SNR is in general so low that single-trial responses cannot be captured for small amplitude early responses. In this study, we were mainly interested in the development of the late cortical activation, and thus the stimulus paradigm was optimized for the late cortical responses. Therefore, the amplitude values are not extracted for the earliest components (middle latency responses and brainstem responses) in humans. All 70 measured epochs were analyzed in animal subjects. In humans, the final number of artifact-free epochs varied, and based on the minimum number of trials in individual subjects, the first 76 epochs were analyzed in all human subjects.

### Statistics

#### Averaged responses

To analyze the averaged responses in humans, we collected both amplitude and latency measures (maximum amplitude and peak latency) separately for each individual in the early (0 to 150 ms, M100 response) and late (150 to 450, M250 response) time windows (Fig. 1). In rats, the amplitude and latency were collected from the same general time windows, by using the following specific time windows for distinct deflection in the early time window: 0 to 12 ms (N6); 0 to 25 ms (P14); 12 to 37 ms (N21); 25 to 75 ms (P41); 50 to 150 ms (N108) and for the late time window: 150 to 350 ms (P214). See also Table 2.

**Table 2:** Time windows (ms) of analyses. Time windows were used for extracting maximum amplitudes and peak latencies for the statistical analysis of single-trial and averaged data in humans and rats.

	**Single trials in rats**	**Averages** **in rats**	**Single trials in humans**
Brainstem	0 to 10	0 to 12	–
Early cortical I	10 to 30	0 to 25 12 to 37	–
Early cortical II	30 to 150	25 to 75 50 to 150	0 to 150
Late cortical	150 to 450	150 to 350	150 to 450

A one-way analysis of variance was used to evaluate the age differences in the strength of averaged auditory responses separately in humans and animals. A post hoc test with Bonferroni correction was used to compare the means of the age groups. The alpha level was *P* < 0.05. Levene’s test of equality of error variance indicated that all of the groups had the same variance.

#### Single trials

For single-trial analysis, the maximum values of evoked deflections were collected from the early (0 to 150 ms) and late (150 to 450 ms) time windows in humans. In rats (for whom more deflections were identifiable also at single-trial level), the early time window was additionally divided to timewindows of 0 to 10 ms, 10 to 30 ms, and 30 to 150 ms, where both negative and positive local maxima were collected. The peak latencies were collected by computing the time points at which the maximum/minimum values were reached in each time window. In humans, the amplitude values reflected the strength of current activity at ~100 ms (M100m) and at ~250 ms (M250), with specific current direction, and it was not reasonable to collect negative values outside these time windows, which may not reflect the same location of activity. In rats, the amplitude values directly reflect the electric potential in specific location (relative to the reference electrode), and thus both positive and negative values were collected from the waveform.

Statistical analyses of single trials were conducted using multilevel, multigroup modeling with pairwise comparisons in Mplus (version 8.4). This approach allowed us to account for the hierarchical nature of the data, where measurements were nested within subjects. The hierarchical organization of the data referred to the fact that each subject had multiple measurements, and the dataset included multiple subjects. The Wald test was used to examine differences in peak latency and amplitude between age groups and time windows, followed by pairwise comparisons. Maximum likelihood estimation with full information (FIML) was applied, which allowed us to include all available data while assuming that any missing data were missing at random.

The statistical model considered 2 levels of variation: Within-level variation represented differences in single-trial responses within each subject (ie variation between repeated measures for the same human or rat) and within-level comparisons thus assessed whether the variability of responses within individual subjects (ie across trials) is consistent or differs across groups. Between-level variation reflected differences in responses between subjects (ie differences between individual humans or rats) and between-level comparisons evaluate whether the overall variability between subjects (ie individual differences) is similar or differs between groups.

To determine the degree of clustering in the data (ie how much of the total variance was explained by differences between subjects versus within subjects), intraclass correlation coefficients (ICCs) were calculated. Low ICC values indicate that most variation occurs within subjects, meaning individual responses vary significantly across trials. High ICC values, on the other hand, suggest that most variation occurs between subjects, meaning individual subjects differ from one another more than their responses vary across trials. Since intraclass correlation (hierarchy within the data, ie the degree to which between-subjects variation accounts for total variance) was detected, a 2-level test was conducted for the latency values. Mean differences of maximum amplitudes in the different time windows across the 3 age groups were tested using a single-level model, focusing on time windows as the unit of analysis. However, the hierarchical structure of the data—where each subject contributed multiple values—was accounted for by adjusting the standard errors and *P*-values to ensure accurate results.

## Results

Figure 1 depicts the time course of the sound-evoked activation (AER) at source level (Fig. 1a and c) and in sensor level (Fig. 1e) in the left hemisphere of each age group. For comparison, Fig. 1b shows the AER in the 3 age groups of rats, as measured with the silver wire electrode from the surface of the primary auditory cortex. The time course indicates 2 separate time windows of activity, at ~100 ms and ~250 ms. Figure 1c visualizes the time course of activation in the vertices of auditory cortex region of interest (ROI) (Heschls gyrus & Planum Temporale) in the left hemisphere, evidencing the robust late-emerging 250-ms activation in the child groups. The ECD model of the source current in each age group, and the distribution of sensors that were used for their localization are depicted in Fig. 1d and e, respectively. We first tested whether the amplitude and timing of the averaged activation in the early and late time windows differed between age groups. We then examined the degree of single-trial variance in response timing for both early and late responses across the different groups, separately for the 2 species. Finally, we assessed whether the relative strength of early vs. late responses in single-trial response amplitude showed age-related differences. The focus was on the late response and its relative magnitude and consistency in reference to early activation. Thus, in the single-trial analysis in rats, we focused on current activation with the same polarity as the late response (P14, P41 vs. P214). The analysis of the opposite polarity is reported in Supplementary Appendix D.

### Averaged late auditory-evoked activation is similarly enhanced in children and juvenile rodents

#### Late auditory activation in humans across different age groups

The late auditory response (M250) peaked on average at 284 ms (latency range for maximum: 193 to 325 ms across all the human subjects). The main effect of age was evident in the amplitude of the response (maximum, [F{2, 30} = 29.85, *P* < 0.001]), and both child groups showed a significantly stronger response in this time window than adults (preadolescents vs. adults, *P* < 0.001; adolescents vs. adults, *P* < 0.001). There was no difference in latency (mean latency for preadolescents at 257 ms, for adolescents at 246 ms and for young adults at 273 ms).

#### Late auditory activation in rats across different age groups

The late auditory response (P214) occurred on average at 214 ms in rats (latency range for maximum: 150 to 338 ms across all animals). As in humans, the main effect of age was evident in the amplitude of the response (maximum, [F{2, 27} = 7.95, *P* < 0.01]) and there were significantly stronger responses in preadolescent rats than in mature rats (*P* < 0.01). In rats, preadolescents showed also higher amplitudes than the adolescent group (*P* < 0.01), which did not differ from the mature group. Furthermore, the main effect of age in the peak latency of the P214 response (F(2, 27) = 6.95, *P* < 0.01) reflected longer response latencies in the preadolescent rats (247 ms) than in the adolescent (194 ms) and adult rats (202 ms) (preadolescents vs. adolescents, *P* < 0.01; preadolescents vs. adults, *P* < 0.05).

### Early transient responses show species-specific differences

#### Early auditory activation in humans

The early auditory response component (M100) peaked on average at 91 ms in adults, at 108 ms in adolescents and at 106 ms in preadolescent children (see Fig. 1a). The M100 response was detected in all adults, in 6 adolescents and in 4 preadolescents. The amplitude of the M100 responses was significantly higher in adults than in children (adolescents and preadolescents grouped together) (*P* < 0.01).

#### Early auditory activation in rats

Neither of the early components (P14, P41) with the same polarity as the late N214 response showed effect of age on the response amplitude (Fig. 1b). The results concerning the rest of the early components (N6, N21 and N108 are given in Supplementary Appendix D).

### Juvenile pattern of AERs is distinguishable at the level of single trials in both humans and rats

**Fig. 3:**
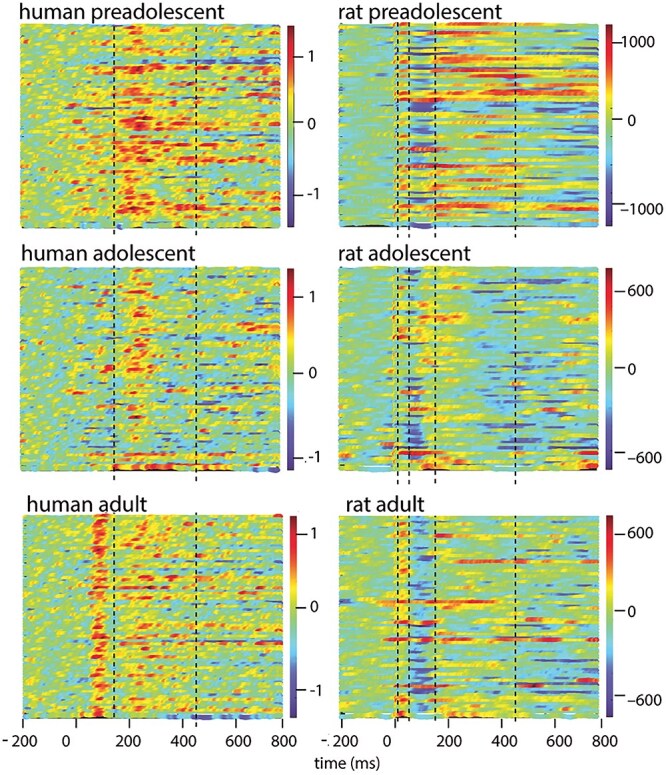
Time-varying amplitudes in single trials in humans and rats. Single-trial amplitude time series of individual subjects’ auditory responses. The response amplitude values are illustrated with color codes across time in the horizontal direction (nAm for humans and µV for rats), and consecutive trials are run in vertical columns starting from the first trial in the uppermost part of each subfigure. Dashed lines depict the time windows used for collecting amplitude and latency values for the single-trial analysis.

Figure 3 depicts the single-trial AERs for 3 human and rat individuals, 1 from each age group (see Supplementary Appendix B for all individuals). Visualization of the individual epochs shows a similar emphasis on activation in the late time window (at ~250 ms) in young individuals (top and middle panels), as was evident in the average responses. In adult/mature individuals, activation at the early stage (at ~100 ms) seems to show more systematic response, hence with reproducible timing across single trials, than later activation. In visual examination, this pattern appears clearer in humans than in rats. For statistical analysis of age-related differences, the maximum values of evoked deflections (maximum amplitude) and the time points when the maximum values were reached (peak latencies) were collected from time windows 0 to 150 ms and 150 to 450 ms in humans and from time windows 0 to 10 ms, 10 to 30 ms, 30 to 150 ms, and 150 to 450 ms in rats.

#### Single-trial timing in humans: responses in the late time window demonstrate more consistent timing in children compared to adults

**Fig. 4:**
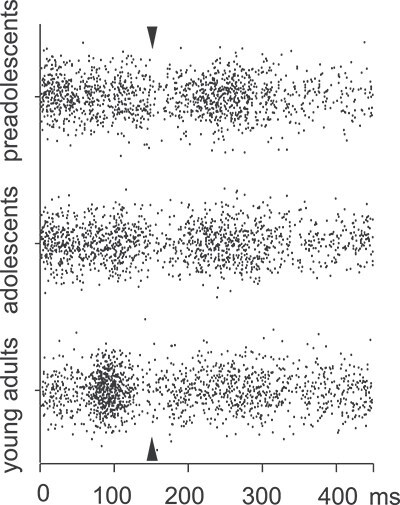
Timing of activation peaks in single trials in humans. The latency distribution of the local amplitude maxima of the single trial evoked responses in humans, extracted in 2 time windows, early (0 to 150 ms) and late (150 to 450 ms). The arrows point to the cut point between the time windows (150 ms).

Figure 4 demonstrates the distribution of the single-trial peak latencies in the 2 time windows in humans. The intraclass correlations (reflecting the degree of the clustered structure in the data) were significant both at the late (9%, *P* = 0.05) and at the early (11%, *P* < 0.01) time windows, indicating that part of the total variance was accounted for by between-subjects variation.

In the late time window, the total variance (ie including variance at within and between levels) of peak latency values differed significantly by age group (W[2] = 17.93, *P* < 0.001). The variance in the preadolescents was smaller than that in the adolescents (*P* < 0.05) and in adults (*P* < 0.001). When this total variance was examined at within (ie single-trial variance in each individual) and between (ie variance between individuals) levels, the age groups differed for within-level comparison (W[2] = 5.93, *P* < 0.05): individuals in preadolescent group had a smaller variance across single-trial values than individuals in the adolescent group (*P* < 0.05). However, the groups did not differ in between-level comparison.

Also, in the early time window, the total variance of peak latencies differed significantly by age group (W[2] = 14.04, *P* < 0.001). The total variance was smaller in adults compared to preadolescents (*P* < 0.001) and adolescents (*P* < 0.001). In between and within levels, comparisons were insignificant.

In sum, at single trials, the consistency of timing of responses was higher for children than adults in the late time window (~250 ms), while it was higher for adults than for children in the early time window (~100 ms). This is evident in Fig. 4, which shows more densely distributed values for preadolescents than for adults in the late time window and the opposite in the early time window.

#### Single-trial timing in rats: age-related differences in the consistency of single-trial timing are less pronounced in rats than in humans

**Fig. 5:**
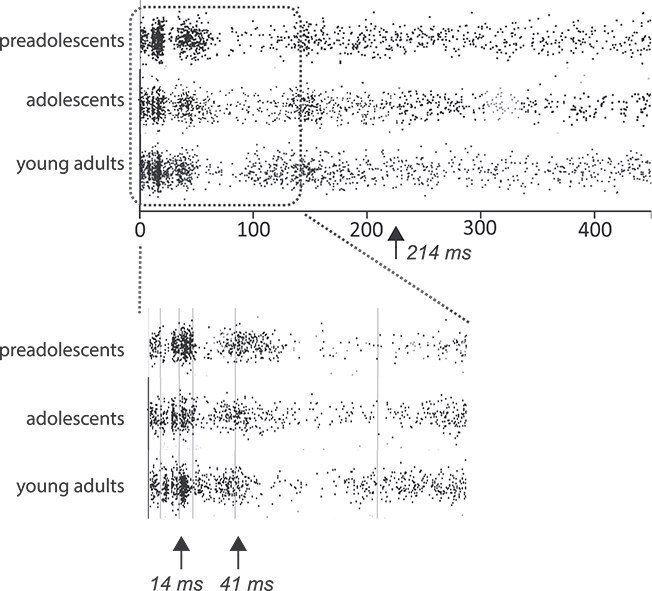
Timing of activation peaks in single trials in rats. The latency distribution of the local amplitude maxima of the single-trial evoked responses in rats, with zoom in to the first 150 ms. The vertical lines and arrows indicate the timing of response maxima in the averaged waveform (for P14, P41, and P214, note that single-trial timing of N6, N21, and N108 is reported in Supplementary Appendix D).

Similar to humans, the local maxima identified in the average waveform were also evident at the level of single trials in rats (see Fig. 5 for the peak latency distribution for positive components, and Appendix D for the negative components). Intraclass correlations were significant for the early peaks at 10 to 30 ms (P14; 33%, *P* < 0.001) and at 30 to 150 ms (P41, 27%, *P* = 0.001), but not for the late peak at 150 to 450 ms (P214, 4%, *P* = 0.10).

The total variance of peak latency values at the late response (P214) did not differ significantly by age group (W = 5.5, *P* = 0.06); with a *P*-value close to the significance level, the absolute level of latency variance was smaller in the preadolescent and adolescent group than in the adult group (similar to the 676 human data). Differences in within and between levels were not significant.

In the early time window the P14 response did not differ in total or within levels, but in between-level variance, there was a significant difference (W = 9.44, *P* < 0.001). The young adults group showed a larger variance than the adolescents group (*P* < 0.01). The P41 response did not have a variance difference in total, yet in within-level variance, there was a significant difference (W[2] = 6.35, *P* < 0.05): the variance across single trials within individuals was larger in adolescents than in preadolescents (*P* < 0.05). In addition, the between-level variance showed a significant difference (W[2] = 10.12, *P* < 0.01), adults showed a larger between-subject variance than adolescents (*P* < 0.01).

In sum, the peak latency values in rats showed more variance than in humans. No consistent developmental differences in rats emerged; however, some pairwise comparisons indicated larger variances in the adult group.

#### Single-trial amplitude variance in humans: unlike adults, children show stronger emphasis on long-latency than on early-latency responses

**Fig. 6:**
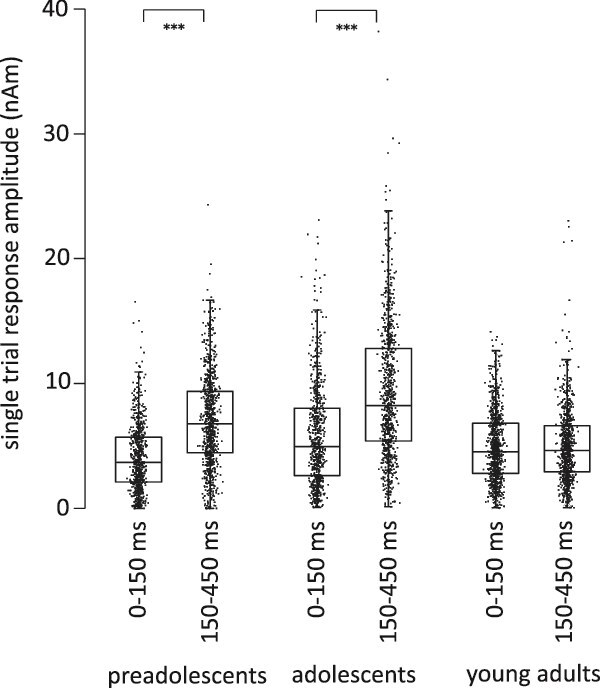
Amplitude of activation peaks in single trials in humans. Maximum amplitude values of individual responses in humans in the early (0 to 150 ms) and late (150 to 450 ms) time windows.

Figure 6 illustrates the results of the complex analysis of the amplitude differences between the time windows in the 3 age groups of humans. Although a similar sequence of auditory responses was consistently demonstrated in subjects across all age groups (encompassing early and late peaks), the emphasis of activation across the time windows seemed to depend on age. The complex method showed significant amplitude differences between time windows (W[2] = 36.58, *P* < 0.001). The single-trial evoked responses showed higher amplitudes in the late time window (150 to 450 ms) than in the early time window (0 to 150 ms) in preadolescents (*P* < 0.001) and adolescents (*P* < 0.001). However, in adults, there was no difference between the early and late time windows in single-trial peak amplitudes.

#### Single-trial amplitude variance in rats: Juvenile rats show a unique emphasis on the long-latency 214 ms response, particularly in comparison to the 41 ms response

**Fig. 7:**
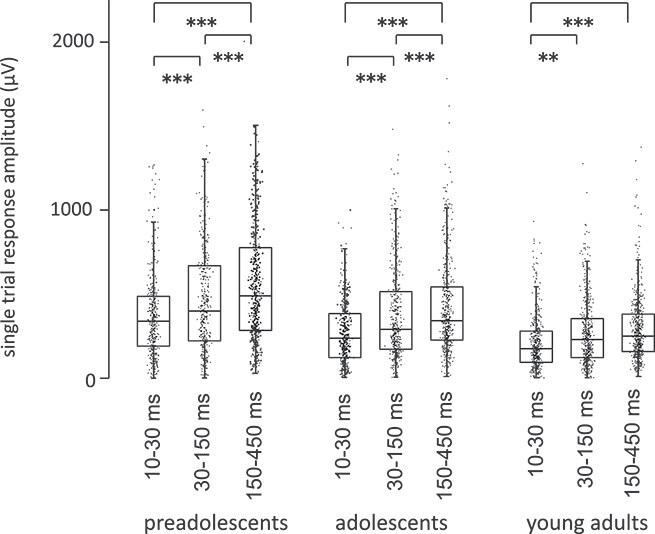
Amplitude of activation peaks in single trials in rats. Single-trial maximum values for P14, P41, and P214, separately in each age group of rats.

Figure 7 illustrates the results of the complex analysis of the amplitude differences between the time windows in the 3 age groups of rats, modeled at a single level. The analysis of the single-trial amplitude values in rats revealed essentially similar results to the comparative analysis in humans.

The maximum single-trial amplitudes for P14, P41, and P214 (Fig. 7a) showed in general significant differences (W[2] = 21.81, *P* < 0.001). In preadolescents, the single-trial peak amplitudes across the 3 time windows differed significantly (W[2] = 39.02, *P* < 0.001): amplitude values were larger for the third (P214) than for the first (P14) response (*P* < 0.001) or the second (P41) response (*P* < 0.001) and also larger for the second than for the first response (*P* = 0.001). Adolescents also showed significant differences (W[2] = 22.73, *P* < 0.001). The amplitudes were larger for the third than for the first response (*P* < 0.001), for the third than for the second response (*P* < 0.001), and for the second than for the first response (*P* < 0.001). Adults had significant differences as well (W[2] = 54.89, *P* < 0.001): the amplitudes were larger for the second than for the first response (*P* = 0.01), for the third than for the first response (*P* < 0.001), but the third response did not differ from the second response.

In summary, also at the single trial level, the P214-ms response amplitudes, as compared with responses at earlier latencies, were pronounced in young rats. In mature rats particularly the difference between P41 and P214 was absent.

## Discussion

We aimed to determine whether an enhanced amplitude of long-latency evoked responses is a characteristic feature of the juvenile auditory cortex across species. Our findings indicate that both human children and young rats exhibit robust long-latency responses, peaking ~200 to 250 ms following simple auditory stimulation, whereas such late activation is attenuated in the mature auditory cortex of both species. Furthermore, single-trial analyses reveal that the delayed cortical responsiveness observed in juveniles is evident at the individual trial level, particularly in humans. The emphasized prolonged evoked activity suggests that the juvenile auditory cortex is characterized by heightened reactivity of its cortical synaptic circuits, particularly at long latencies.

Considering the significant phylogenetic distance between humans and rats, it is surprising that the age-related differences in early and late cortical responses to auditory stimuli resemble each other in the 2 species. Preadolescent rats exhibited stronger late responses than mature rats, with these responses occurring on average at absolute latencies close to those observed in humans (at 214 ms in rats and 250 ms in humans). Our current experimental paradigm does not allow for a direct one-to-one correspondence of evoked response components between species. However, some previous studies suggest that while early responses are systematically faster in rats, for the later responses (such as the P3 component) the difference in timing between species becomes less evident (Sambeth et al. 2003).

There were also important differences between species. In humans the adolescent group showed similar amplitude values to the preadolescents, while in rats the response in adolescent rats showed amplitude values close to mature individuals. This may be due to the faster maturational trajectory in rodents than in humans, even though we aimed at matching the maturational stage. It is however, important to note that the experimental paradigms were not identical, limiting direct comparisons across species. This study’s paradigm aimed to explore whether, when comparing late versus early responses, the late responses are more pronounced in younger individuals, and to determine whether this phenomenon is observed across species. Further studies should elaborate to what extent the developmentally specific response in the two species reflect comparable functional properties in the two species.

In humans, a prolonged activation pattern has been reported also earlier (Ponton et al. 2000; Sussman et al. 2008; Parviainen et al. 2019), but the emphasis has been on cognitive processing or processing of different perceptual categories. In electrophysiological recordings of rodents on the other hand, the focus has been on the early responses, and the epoch of analysis is typically limited to the first tens of milliseconds. This is justified in studies of passive sensory processing, as the neural signal progresses rapidly beyond primary auditory areas. Indeed, after 100 ms, the evoked activation is usually associated with cognitive aspects, sensory integration, or top-down processing of the stimuli that are less frequently approached in nonhuman species. This convention may, however, leave unnoticed developmentally meaningful slower sensory responses. Although the sensory pathway transmits the signal rapidly to higher level areas in the juvenile brain, it may well be that, in parallel, developing sensory cortices exhibit recurrent synaptic activity: thus, later response does not necessarily mean “higher level” in processing. This type of prolonged electric discharge in early sensory areas may be important to enable the formation of associations (hence learning) within a local and especially cortico-cortical network, therefore indicating the level of cortical plasticity.

Central to our interpretations in the present study is that the late response was evoked in purely passive conditions without attention, priming, or oddball manipulation. The age-related differences must thus be considered as indicating changes in cortical responsivity to external stimulation, hence, properties of the synaptic signaling pathway. The few studies that examined the behavioral significance of these passive response properties have connected stronger or longer-lasting activation in this time window with lower level of automatization or efficiency in cognitive tasks (Albrecht et al. 2000; Parviainen et al. 2011). However, other studies have linked stronger M250 responses with improved cognitive performance, for example, better phonological skills in the case of specific language impairment (van Bijnen et al. 2019) or higher response inhibition (van Bijnen et al. 2022). It is thus not straightforward to conclude whether increased activity in this time window should be considered behaviorally beneficial or an indicator of an immature and hence less efficient system. A thorough understanding of the developmental changes in the response properties of the cortical auditory pathway is essential for elucidating the neural basis of higher level neurocognitive development.

We showed emphasis on late versus early activation in the juvenile cortex also at the level of individual trials, albeit this result was clearer in humans than in rats. In human preadolescents and adolescents, the single-trial amplitudes were consistently stronger in the late (>200 ms) than in the early (<200 ms) time window. Also preadolescent and adolescent rats showed emphasized amplitudes for the responses in long-latency versus middle-latency time windows (ie P214 vs. P41). This difference between time windows in the single-trial response amplitudes seemed to vanish along with development and was not present in mature subjects (either in humans or in rats). In rats, however, the higher signal quality enabled examination of single-trial variance even in the early components, and young and older rats did not differ in their overall tendency to exhibit higher amplitudes for the late (P214) responses compared to the very early (P14) responses.

In addition to the emphasized amplitude of the late M250/P214 response in juvenile individuals, the single-trial analysis in humans indicated greater consistency in the timing of activation during the late time window. The variation in the timing of the M250 responses appeared smaller in preadolescent children than in adolescents or adults. Given the more consistent timing of single-trial responses for later (M250) compared to earlier (M100) components, it seems unlikely that volatile temporal precision in synaptic information processing underlies the late evoked response component. On the contrary, when probed with simple auditory stimuli, the juvenile auditory cortex systematically exhibits a prominent, sluggish response, likely reflecting distinct synaptic signaling and unique response properties of the juvenile cortex. It is important to note that the comparison was performed between these 2 response types and does not reveal the inter-trial consistency in the timing of other response components. In children before school age, a P/M50 response is often reported with latencies closer to 100 ms in children, overlapping with the emerging N/M100 response. With roughly opposite polarity in current direction, these 2 responses clearly reflect distinct generators. We included current direction in the source model which allowed us to selectively focus on M100 response. Whether the developmental decrease in the latency of the P/M50 response is associated with changes in temporal precision remains to be investigated in future studies.

The age-related differences in single-trial amplitude levels and timing consistency were less pronounced in rats than in humans. In rats, mature individuals occasionally exhibited responses in the late time window. This finding may be related to fluctuations in ongoing brain states that are likely to influence evoked activity (Civillico and Contreras 2012). In general, in the case of low amplitude signals, the timing of single-trial responses may also reflect random variation, and it is thus difficult to determine whether to interpret the findings as low SNR or high variance in response latency. The same challenge also holds for determining the M250 response in human adults and for the M100 response in human children at single trial level.

The focus in the present study was on late activation and the relative emphasis between late vs. early amplitudes. The results however allow some discussion also on early time window. The early transient response components showed stronger species-specific differences. This was to be expected from the extensive literature on auditory cortical responses in each species. In humans, in line with previous studies (Hillyard and Picton 1987); Parviainen et al. 2005), adults exhibited a prominent M100 response, which was less clear in younger groups. Interestingly, the single-trial analysis in humans revealed that the temporal consistency of activation (ie the intertrial precision of signaling) in the M100 response was also higher in adults than in children. In rats, the higher signal-to-noise ratio (and focal measurement from the brain surface) enabled the identification of the more detailed canonical sequence of AERs before the prolonged pattern. Compared to the late response, the early responses showed, in general, more consistent latencies at the single-trial level. Overall, the early responses seemed to reach mature characteristics at an earlier age than the late prolonged pattern since there were no differences in the amplitudes between age groups for most of the early responses. As an exception, the N21 response was enhanced, both in averaged waveform and in single-trial analysis, in the preadolescent rats compared to the adolescents. However, since the stimulus conditions were not designed to compare early latency differences by age or species, we cannot conclude that there are no age-related changes in auditory response timing in rodents after 28 d.

The passively presented simple tones used in this study can be regarded as probes for examining the response properties of the cortical auditory pathway. Our results demonstrate that the synaptic response properties—generally assumed to underlie cortically measured evoked responses—differ significantly in the developing brain, even at the age of 13 in humans. Based on our current findings, we propose that the unique prolonged response observed in the juvenile brain reflects increased reactivity of cortical synaptic circuitry to external stimulation in children. Interestingly, when cortical reactivity is probed with TMS rather than natural sensory stimuli, the sensorimotor cortex in children exhibits prolonged reactivity similar to the M250 response observed in our study (Määttä et al. 2017). Together with previous findings in the sensorimotor cortex and the prolonged activation pattern evidenced in the somatosensory cortex of infants (Pihko et al. 2009), our results imply that a domain-general, and possibly species-general, delayed response type may emerge in sensory cortices—a characteristic feature of the maturing cortex.

What could be the underlying mechanism at the synaptic level for this prolonged activity? The recorded magnetic or electric field changes measured outside the brain are directly associated with the local field potentials that arise from synaptic signaling in the laminar circuits. Thus, any developmental change in synaptic connectivity is likely to be reflected in the MEG/EEG fields, albeit with less spatial resolution and only as a rough sum of the more detailed laminar-level events. However, importantly, the temporal characteristics reflect the electrical events associated with synaptic signaling, and it is the developmental changes in these postsynaptic currents that most likely give rise to the observed age-related differences.

Although excitatory feedforward connections are the main driveway for information transfer, protracted development of inhibitory circuits brings about precision and “computational power” to the circuitry, for example, by narrowing the synaptic integration window in the auditory cortex (Oswald and Reyes 2011). The temporal properties of the inhibitory neurotransmitter receptors, which can be either fast ionotropic (GABA-A) or slower metabotropic (GABA-B) (Connor et al. 1988; MacDonald and Olsen 1994; Couve et al. 2000), provide a relevant source of electric activity considering the developmental changes in the cortically recorded responses. Using pharmacological manipulation and TMS, the evoked responses during the first 30 ms were suggested to reflect excitatory neurotransmission, whereas later peaks at 40 to 50 ms and >100 ms were associated with inhibitory neurotransmission either by GABA-A or GABA-B receptors, respectively (Ferreri et al. 2011; Premoli et al. 2014).

Temporal characteristics of GABAergic synapses are the same across cortical areas and presumably also across species (Bettler et al. 2004). Indeed, based on its temporal characteristics, the prolonged response in the juvenile auditory cortex could be related to developmental changes in GABA-B neurotransmission. In support of this interpretation, a few TMS-EEG studies in children demonstrate a later component at 200 to 300 ms, which differs both in amplitude and in spatial distribution from the adult response. This later child-specific activation was suggested to reflect developmental differences in GABA_B_ergic neurotransmission and/or in cortico-subcortical–cortical loops (Ferreri et al. 2017; Määttä et al. 2019). We thus propose that the late emerging activity in the auditory cortex in the present study reflects increased reactivity of the synaptic circuitry in the juvenile state, possibly associated with modulations in GABAB-related neurotransmission. However, further studies are needed to directly test the role of GABAergic signals in cortically measured evoked fields. The speedup of the response latencies, especially in the early time window, could also reflect the influence of myelination, besides increased temporal precision of synaptic signaling along with the maturation of the GABA(A)ergic system.

Thus, the prolonged response in children could indicate increased reactivity of the cortical circuitry at these long latencies. These juvenile stage responses may indicate decreased levels of inhibition, perhaps due to the well-established protracted developmental trajectory of inhibitory GABAergic synaptic signaling (Le Magueresse and Monyer 2013), specifically demonstrated for the auditory cortex by Takesian et al. (2010).

Several factors need to be acknowledged when interpreting the current findings. The 2 species were not recorded under identical conditions: for humans, we used passive presentation of stimuli during the awake state, whereas the rats were anesthetized. However, the passive response properties of the cortex are assumed to be largely unaffected by light anesthesia, and more importantly, the age-group comparisons within each species should not be influenced by differences in measurement conditions. Direct comparisons between species are further compromised because the responses were collected under passive conditions without task manipulations that could, in the future, help confirm comparable response types across species. As a general framework, previous studies provide a rough timeline for the correspondence between rat and human evoked response components, indicating that temporal differences are greater at early latencies and decrease with longer response latencies. Nevertheless in the current study, it is possible that the neurobiological phenomena underlying the recordings—and the observed age-related differences—are not the same in the 2 species.

Although ECoG and MEG share the same underlying neural generators, the methods used to estimate the strength and timing of current activity differ between species, and the anatomical location of the auditory cortex relative to the recording surface also varies. Thus, we did not aim to match polarities across species. In rats, where electric potential differences are recorded directly, polarity depends on the reference electrode. In humans, we focused on robust current activity originating from the supratemporal auditory plane, which exhibited a maximal dipolar pattern and a current direction toward deeper cortical layers at ~250 ms in children and ~100 ms in adults. Although the overall spatial estimates were highly similar between age groups, the timing of current response extraction naturally varied, which may have influenced amplitude estimates. Indeed, it was not possible to extract a reliable dipolar current estimate from the late activation pattern in adults, as the amplitude of this response was close to the noise level in many individuals. Importantly, the fact that sensor-level raw data revealed similar amplitude differences in the late time window suggests that using different time windows in the source model did not affect the observed age-group differences.

Finally, although at a juvenile stage, the current study focused on relatively old subjects in both species, and the developmental changes in children under 9 yr or rodents under 1 mo of age need to be explored in future studies.

To conclude, we provide evidence of the maturational steps of the late auditory activation in another mammalian species alongside data from humans, which confirms the hypothesis of the obligatory nature of the late activation in the developing brain. The findings at the level of single trials confirm that the enhanced and delayed activity recorded from outside the cortex both in juvenile humans and in rats reliably reflects response properties of the developing neural circuitry, rather than being a result of the averaging procedure. In fact, the late activation was not only evident at the single-trial level but also showed more consistent trial-by-trial timing in human preadolescent children compared to the earlier, adult-like 100-ms response. This implies that the late evoked activation does not reflect imprecise temporal accuracy in the circuitry but represents a separate prolonged synaptic event, which is likely to be meaningful and functional for the developing brain*.*

## Supplementary Material

CerCor-2023-00248_SupplementaryMaterial_Resubmission_080925_bhaf274

AppendixA_240925_bhaf274

AppendixD_240925_bhaf274
