# Supplementary material for: Long-latency auditory evoked responses across species show increased amplitude during early life
Source: Cereb Cortex. 2026 Jan 7;36(1):bhaf274. doi: 10.1093/cercor/bhaf274 (PMC12774839; doi:10.1093/cercor/bhaf274)
Supplement: CerCor-2023-00248_SupplementaryMaterial_Resubmission_080925_bhaf274 [file cercor-2023-00248_supplementarymaterial_resubmission_080925_bhaf274.pdf]

Supplementary material

Long-latency auditory evoked responses across species show increased amplitude during early life

Running title: Auditory cortex development in humans and rats

Krista Lehtomäki,[1] Jari Keinänen,[1] Lauri Parkkonen,[2] Riaz Uddin Mondal,[3,4] Markku Penttonen,[1] and

Tiina Parviainen[1,5,*]

[1] Center for Interdisciplinary Brain Research & Department of Psychology, University of Jyväskylä, 40014 Jyväskylä, Finland

[2] Department of Neuroscience and Biomedical Engineering, School of Science, Aalto University, 00076 Aalto, Espoo, Finland

[3] Faculty of Information Technology, University of Jyväskylä, 40014 Jyväskylä, Finland

[4] Department of Information & Communication Engineering, Faculty of Engineering, University of Rajshahi, Rajshahi 6205, Bangladesh

[5] Lead contact

*Corresponding author: tiina.m.parviainen@jyu.fi
Address: Department of Psychology, University of Jyväskylä, PO BOX 35, FI-40014, University of Jyväskylä, Finland; tel. +358 400 856 423; email: tiina.m.parviainen@jyu.fi

Appendix A. Average responses to sine-wave tones in rats.

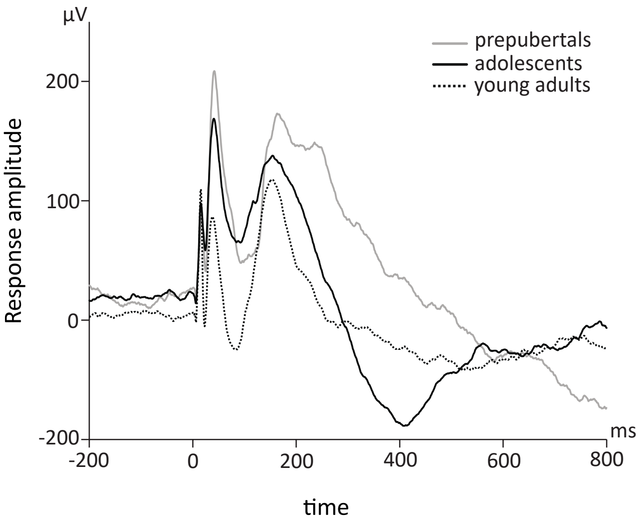

Appendix B. The number of single trials in each age group and single-trial time series in all individuals for humans and rats

Table B1. Number of single trials (min, min – max) in each age group in humans and rats

	preadolescents	adolescents	young adults
Humans			
M100 (N)	69, (64-73)	70, (61-73)	75, (73-76)
M250 (N)	74, (72-76)	74, (70-76)	74, (70-76)
Rats			
N6 (N)	38, (30-44)	37, (31-34)	38, (31-41)
P14 (N)	43, (26-54)	42, (35-61)	46, (39-61)
N21 (N)	48, (34-62)	41, (34-53)	48, (39-55)
P41 (N)	42, (29-51)	47, (37-57)	51, (40-64)
N108 (N)	47, (37-56)	46, (33-56)	53, (44-63)
P214 (N)	48, (34-56)	52, (43-65)	56, (43-66)

Figure B1. Single-trial time series in all individuals for humans and rats

A humans

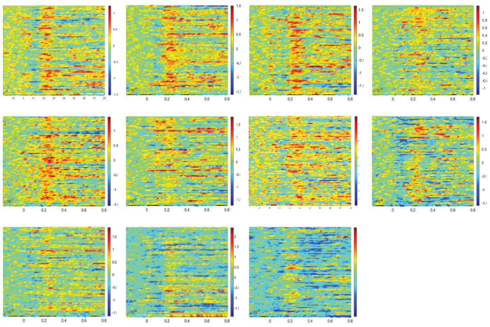

A rats

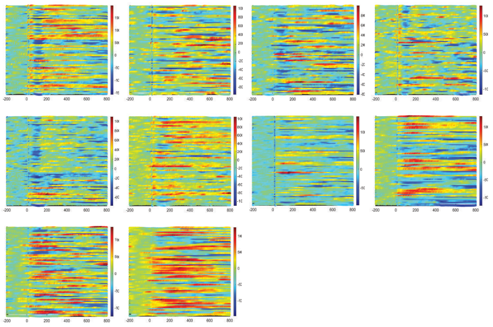

B humans

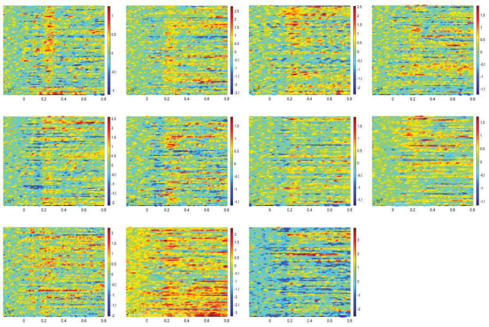

B rats

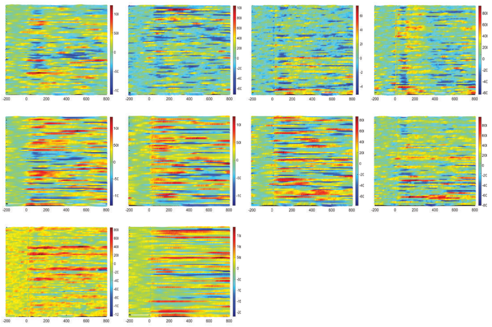

C humans

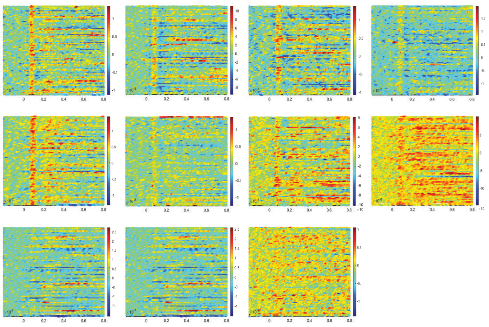

C rats

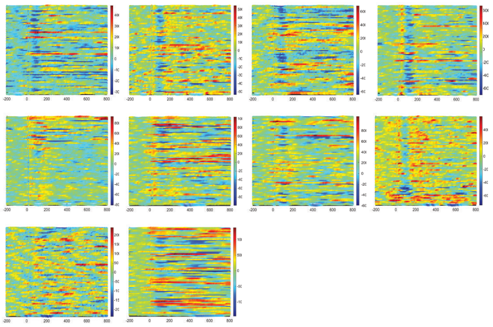

Appendix C. MNE Source analysis methods

To illustrate activation differences across age groups independently of the source modeling approach, we conducted an MNE source analysis on human data (see Figure 1c). The data were filtered with a 0.1 Hz high-pass and 40 Hz low-pass filter. Blink and heartbeat artifacts were removed using ICA, and epochs from -200 to 800 ms (baseline corrected to -200 to 0 ms) were extracted. The source space comprised 4,098 active vertices per hemisphere, located at the gray-white matter boundary. The normalized covariance matrix, used for whitening, was calculated individually from epoch baselines (-200 to 0 ms). For the inverse operator, a loose orientation constraint (0.2) and depth weighting (0.8) were applied. In the final source estimate, source orientation was set normal to the white matter boundary to maintain the directionality of the activity. Regions of interest (ROIs), including Heschl's gyrus, sulcus, and planum temporale, were defined based on the Destrieux atlas in each hemisphere. Templates from the McGill pediatric atlases and the Fsaverage template were used to construct cortically constrained source estimates for the children and adult groups, respectively. Each subject's data were morphed to their respective age-appropriate template (children aged 7-11 years for preadolescents, children aged 10-14 years for adolescents and Fsaverage for adults). Templates were aligned to individual head shapes using fiducial landmarks and additional head shape points digitized during data acquisition. When additional digitized points were unavailable, alignment was based solely on fiducial points. A single-layer boundary element model (conductivity: 0.3) based on the inner skull surface was created for each individual.

Appendix D: Results for the analysis of the averaged auditory evoked responses and single trial activation pattern for the N6, N21 and N108 components in rats.

Early auditory activation pattern in rats: Averaged responses

The first distinguishable component at around 6 ms (N6, at a latency range of 3–7 ms across all animals) showed no differences between the age groups in the maximum amplitude. The following component at around 21 ms (N21, at a latency range of 13–26 ms across all animals) showed a main effect of group in response amplitude ($F(2, 27) = 8.50$, $p = .001$), being stronger in the preadolescent rats than in both the adolescent ($p < .01$) and adult rats ($p < .01$). In sum, only the 21-ms response evidenced age-group differences, with stronger amplitude in the youngest age group than in the two older ones.

Early auditory activation in rats: Single-trial timing

Intraclass correlations were significant for the peaks at 10–30 ms (44%, $p < .001$) and at 30–150 ms (14%, $p < .01$), but not for the peak at 0–10 ms (9%, $p = .11$). For the N6 response, there were no total, within-, or between-level differences in the variance of the peak latency values. For the N21 response, the variance at the total or between level did not differ. At the within level, there was a significant group difference ($W(2) = 10.72$, $p < .01$): in adolescents, the internal variance was larger than in preadolescents ($p = .001$). The N108 response latency showed a significant variance difference in total ($W(2) = 6.02$, $p < .05$), and pairwise comparisons indicated that there was significantly larger variance in adults than in preadolescents ($p < .05$). Also in between-level variance, the difference was significant ($W(2) = 7.2$, $p < .05$): adults had a larger variance than preadolescents ($p < .01$) or adolescents ($p = .01$). There were no significant within-level differences.

Early auditory activation in rats: Single-trial strength

Analyzing single-trial maximum amplitudes of N6, N21, and N108 showed significant differences (W(2) = 40.45, $p < .001$) (Figure 7b). In preadolescents, a single-level test for the time window showed significant differences (W(2) = 165.35, $p < .001$), and the amplitudes of the responses were larger in the third than in the first time window ($p < .001$) and in the second than in the first time window ($p < .001$). There were no differences between the amplitudes in the second- and third-time windows. In adolescents, significant differences were shown (W(2) = 75.6, $p < .001$), and amplitudes in the third and second time windows were larger than in the first time window (3 vs. 1, $p < .001$; 2 vs. 1, $p < .001$), and amplitudes in the third time window were larger than in the second time window ($p < .001$). In adults, significant differences were also shown (W(2) = 59.22, $p < .001$), and comparisons by time windows showed similar results to those in the adolescent group. The amplitudes were larger in the second and third time windows than in the first (2. vs. 1., $p < .001$; 3. vs. 1., $p < .001$) and in the third time window than in the second ($p = .001$). In summary: In preadolescents, the amplitudes of the N21-ms and N108-ms did not differ, while the older groups had smaller N21-ms amplitudes compared to N108-ms amplitudes.

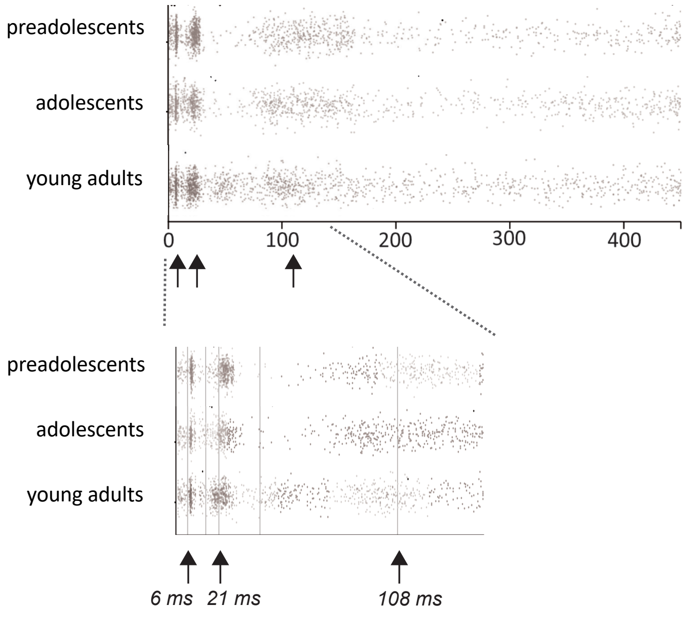

Figure D1. Timing of activation peaks in single trials in rats for the responses at 6 ms, 21 ms and 108 ms. The latency distribution of the local amplitude maxima of the single-trial evoked responses in rats, with zoom in to the first 150 ms.

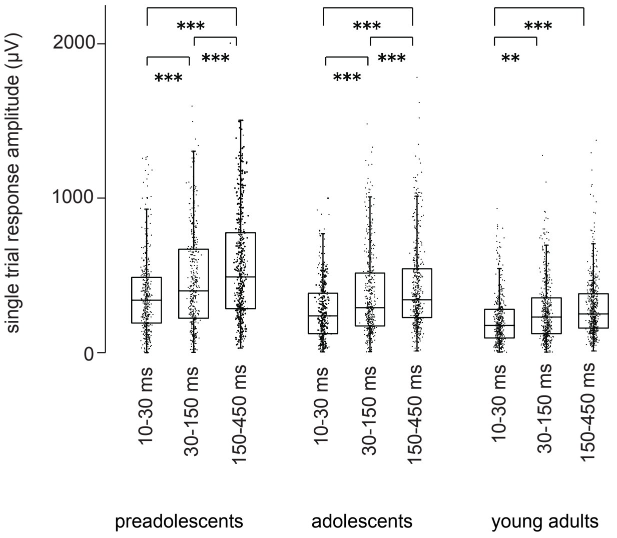

Figure D2. Amplitude of activation peaks in single trials in rats for the responses at 6 ms, 21 ms and 108 ms.
