# Supplementary figures and images for: Long-latency auditory evoked responses across species show increased amplitude during early life
Source: Cereb Cortex. 2026 Jan 7;36(1):bhaf274. doi: 10.1093/cercor/bhaf274 (PMC12774839; doi:10.1093/cercor/bhaf274)

## Appendix A. Average responses to sine-wave tones in rats.

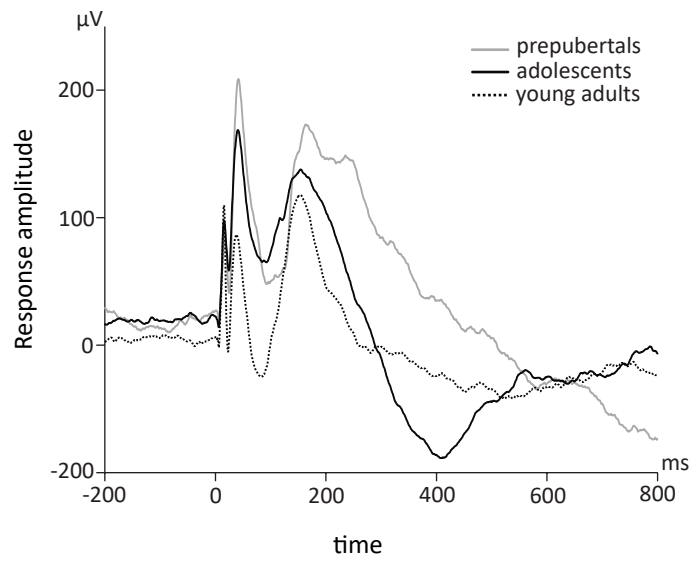

Supplement: AppendixA_240925_bhaf274 [file appendixa_240925_bhaf274.pdf]
