# Supplementary material for: Long-latency auditory evoked responses across species show increased amplitude during early life
Source: Cereb Cortex. 2026 Jan 7;36(1):bhaf274. doi: 10.1093/cercor/bhaf274 (PMC12774839; doi:10.1093/cercor/bhaf274)
Supplement: AppendixD_240925_bhaf274 [file appendixd_240925_bhaf274.pdf]

**Appendix D. MNE Source analysis methods**

To illustrate activation differences across age groups independently of the source modeling approach, we conducted an MNE source analysis on human data (see Figure 1c). The data were filtered with a 0.1 Hz high-pass and 40 Hz low-pass filter. Blink and heartbeat artifacts were removed using ICA, and epochs from -200 to 800 ms (baseline corrected to -200 to 0 ms) were extracted. The source space comprised 4,098 active vertices per hemisphere, located at the gray-white matter boundary. The normalized covariance matrix, used for whitening, was calculated individually from epoch baselines (-200 to 0 ms). For the inverse operator, a loose orientation constraint (0.2) and depth weighting (0.8) were applied. In the final source estimate, source orientation was set normal to the white matter boundary to maintain the directionality of the activity. Regions of interest (ROIs), including Heschl's gyrus, sulcus, and planum temporale, were defined based on the Destrieux atlas in each hemisphere. Templates from the McGill pediatric atlases and the Fsaverage template were used to construct cortically constrained source estimates for the children and adult groups, respectively. Each subject's data were morphed to their respective age-appropriate template (children aged 7-11 years for preadolescents, children aged 10-14 years for adolescents and Fsaverage for adults). Templates were aligned to individual head shapes using fiducial landmarks and additional head shape points digitized during data acquisition. When additional digitized points were unavailable, alignment was based solely on fiducial points. A single-layer boundary element model (conductivity: 0.3) based on the inner skull surface was created for each individual.
